# Clinicopathological features and prognosis of idiopathic membranous nephropathy with thyroid dysfunction

**DOI:** 10.3389/fendo.2023.1133521

**Published:** 2023-03-16

**Authors:** Peiheng Wang, Shulei Wang, Bo Huang, Yiming Liu, Yingchun Liu, Huiming Chen, Junjun Zhang

**Affiliations:** ^1^ Department of Nephrology, The First Affiliated Hospital of Zhengzhou University, Zhengzhou, Henan, China; ^2^ Research Institute of Nephrology, Zhengzhou University, Zhengzhou, Henan, China

**Keywords:** thyroid function, clinicopathological features, prognosis, propensity score matching, idiopathic membranous nephropathy

## Abstract

**Background:**

Thyroid dysfunction is common in patients with kidney disease. However, the relationship between thyroid dysfunction and idiopathic membranous nephropathy (IMN) remains unclear. This retrospective study aimed to investigate the clinicopathological characteristics and prognosis of patients with IMN and thyroid dysfunction compared to patients with IMN and without thyroid dysfunction.

**Methods:**

A total of 1052 patients with IMN diagnosed by renal biopsy were enrolled in this study, including 736 (70%) with normal thyroid function and 316 (30%) with abnormal thyroid function. We analyzed the clinicopathological features and prognostic data between the two groups, using propensity score matching (PSM) to reduce the bias. Logistic regression analysis was performed to investigate the risk factors for IMN combined with thyroid dysfunction. Kaplan-Meier curves and Cox regression analysis were used to evaluate the association between thyroid dysfunction and IMN.

**Results:**

Patients with IMN and thyroid dysfunction exhibited more severe clinical features. Female sex, lower albumin level, higher D-dimer level, severe proteinuria, and decreased estimated glomerular filtration rate were predictors of thyroid dysfunction in patients with IMN. After PSM, 282 pairs were successfully matched. Results from the Kaplan-Meier curves indicated that the thyroid dysfunction group had a lower complete remission rate (*P* = 0.044), higher relapse rate (*P* < 0.001), and lower renal survival rate (*P* = 0.004). The multivariate Cox regression analysis revealed that thyroid dysfunction was an independent risk factor for complete remission [hazard ratio (HR) = 0.810, *P* = 0.045], relapse (HR = 1.721, *P* = 0.001), and composite endpoint event (HR = 2.113, *P* = 0.014) in IMN.

**Conclusions:**

Thyroid dysfunction is relatively common in patients with IMN, and the clinical indicators are more severe in these patients. Thyroid dysfunction is an independent risk factor for poor prognosis in patients with IMN. More attention should be paid to thyroid function in patients with IMN.

## Introduction

Idiopathic membranous nephropathy (IMN) is one of the most common types of renal pathology in adults with nephrotic syndrome and one of the leading causes of end-stage renal disease (ESRD) (1). Massive proteinuria and edema are hallmark clinical characteristics of IMN, and the predominant histopathological alterations include basement membrane thickening and subepithelial immune complex deposition (2). Sixty percent of patients with untreated IMN experience a gradual decline in renal function, and about 35% progress to ESRD within ten years (3). M-type phospholipase A2 receptor (PLA2R) is the main antigen expressed on the podocyte surface of IMN, which exists in 70%-80% of IMN cases (4, 5). Serum PLA2R autoantibody is highly specific for the diagnosis of IMN, and its level is closely related to disease severity, which plays a crucial role in predicting treatment response and disease activity (6–8). There is a close relationship between thyroid hormones and the kidney. Thyroid hormones play an essential role in the renal structure, blood perfusion, glomerular filtration, tubular function, and water-electrolyte balance. Furthermore, the kidney is not only a target organ for thyroid hormones but is also involved in the metabolism and elimination of thyroid hormones (9). Thyroid dysfunction has been reported in different glomerular diseases and increases the risk of developing chronic kidney disease (CKD) in the elderly (10, 11). However, the significance of thyroid dysfunction in the development and prognosis of IMN remains unclear. Therefore, this study analyzed the clinicopathological and prognostic data of patients with thyroid dysfunction and IMN in a large cohort to evaluate the relationship between thyroid function and IMN.

## Materials and methods

### Study participants

From January 2015 to December 2019, 1052 patients with biopsy-proven IMN from the First Affiliated Hospital of Zhengzhou University were included in this retrospective cohort analysis. The following inclusion criteria were applied: (1) complete baseline data and follow-up of ≥ 6 months; (2) age ≥ 18 years; (3) no previous history of thyroid disease; and (4) no glucocorticoids or immunosuppressants before the renal biopsy. Patients with other glomerular diseases, such as IgA nephropathy, diabetic nephropathy, minimal change disease, and/or secondary conditions, such as hepatitis, systemic lupus erythematosus, psoriasis, malignancy, serious infectious diseases, or severe cardiopulmonary diseases, were excluded. Blood was collected within 48 hours of admission to assess the free thyroxine (FT4), free triiodothyronine (FT3), and thyroid-stimulating hormone (TSH) levels. The patients were divided into euthyroid and thyroid dysfunction groups according to the thyroid hormone levels. Patients in the thyroid dysfunction group were further divided into three subgroups: hypothyroid, hyperthyroid, and non-thyroid illness syndrome (NTIS) groups.

This study followed the standards of the Helsinki Declaration and was supported by the Ethics Review Committee of the First Affiliated Hospital of Zhengzhou University (approval number:2022-KY-1187-002). Informed consent was waived owing to the retrospective nature of this study.

### Data collection

Demographic and clinical data were collected at the time of renal biopsy, including age, sex, blood pressure, blood urea nitrogen (BUN), serum creatinine (SCr), uric acid (UA), triglycerides (TG), total cholesterol (TC), blood white blood cells (WBC), hemoglobin, platelets, albumin, estimated glomerular filtration rate (eGFR), M-type phospholipase A2 receptor (PLA2R) antibody, D-dimer, proteinuria, FT3, FT4, and TSH. FT3 and FT4 levels were measured using a commercial radioimmunoassay (RIA) kit (Roche Diagnostics, Mannheim, Germany). TSH levels were measured using a commercial RIA kit (Immunotech, Marseille, France). Two experienced renal pathologists diagnosed the renal biopsy specimens using light microscopy, electron microscopy, and immunofluorescence. Pathological changes were classified into two grades based on the presence or absence of glomerulosclerosis, crescents, mesangial cell proliferation, renal tubular atrophy, renal interstitial fibrosis, inflammatory cell infiltration, and renal arteriolar lesions. Follow-up data included time, proteinuria, SCr, eGFR, albumin, and medications, such as renin-angiotensin-aldosterone system inhibitor (RAASi), corticosteroids, and immunosuppressants.

### Outcomes and definitions

The endpoint event was a composite of SCr doubling, eGFR decrease of >40% from baseline, ESRD, or death from kidney failure (12–14). ESRD was defined as eGFR <15 mL/min/1.73 m^2^ or the need for renal replacement therapy (dialysis or kidney transplantation). Follow-up time was defined as the interval from renal biopsy to the occurrence of the endpoint event or the last outpatient visit. Treatment response included clinical remission and relapse. Clinical remission included complete remission (CR) and partial remission (PR). CR was defined by proteinuria <0.3 g/d, serum albumin >35 g/L, and SCr stability. PR was defined as proteinuria <3.5 g/d with a >50% reduction from baseline. After clinical remission, the reappearance of proteinuria >3.5 g/d was defined as relapse. The reference ranges of FT3, FT4, and TSH levels were 3.28–6.47 pmol/L, 7.9–18.4 pmol/L, and 0.34–5.6 μIU/mL, respectively. NTIS, also known as euthyroid sick syndrome, includes low T3, low T4, low T3 and low T4, high T4, and other abnormalities (15). The time course of kidney disease was defined from discovery to renal biopsy. Hypertension was defined as systolic pressure ≥140 mmHg, diastolic pressure ≥90 mmHg, or the use of antihypertensive drugs. The mean arterial pressure was equal to one-third systolic pressure plus two-thirds diastolic pressure. Nephrotic syndrome was defined as proteinuria >3.5 g/d and serum albumin <30 g/L. The eGFR was calculated using the Chronic Kidney Disease Epidemiology Collaboration (CKD-EPI) equation (16).

### Statistical analysis

Continuous variables with normal distribution were expressed as mean ± standard deviation, and the independent samples t-test, one-way analysis of variance, and Bonferroni method were used for comparisons between the groups. Data that did not follow the normal distribution were expressed as median and interquartile ranges (25%, 75%), and the differences were compared using the Mann-Whitney U test or Kruskal-Wallis test. Categorical variables were expressed as frequency (percentage) and compared between groups using the χ^2^ test or Fisher exact test. Logistic regression was used to analyze the risk factors of thyroid dysfunction in patients with IMN. To reduce confounders and balance baseline variables, we applied propensity score matching (PSM) (17). Matching was performed in a 1:1 ratio using the nearest neighbor approach with no replacement and a matching tolerance of 0.02. The covariates entered into the propensity score model included age, sex, hemoglobin, platelets, albumin, BUN, SCr, UA, TC, TG, eGFR, WBC, PLA2R antibody, D-dimer, proteinuria, and treatment. To compare the rates of CR, relapse, and renal survival between the groups, the Kaplan-Meier curve and log-rank test were used, as well as multiple testing with a Bonferroni correction method. The association between thyroid dysfunction and IMN prognosis was investigated using Cox regression analysis. SPSS version 26.0 (IBM Corp, Armonk, NY, USA) and GraphPad Prism version 8.0.2 (GraphPad Software, San Diego, CA, USA) software were used for statistical analysis and figure creation. All tests were two-sided. A *P* value <0.05 indicated statistical significance.

## Results

### Baseline characteristics

**Figure 1:**
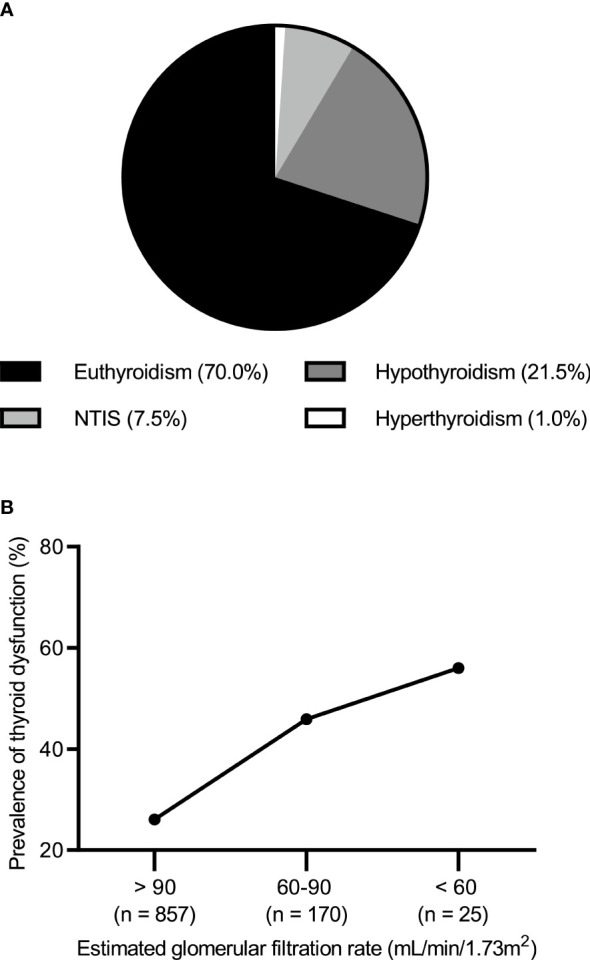
**(A)** Prevalence of thyroid dysfunction in idiopathic membranous nephropathy. **(B)** Prevalence of thyroid dysfunction by the level of estimated glomerular filtration rate.

Of the 1052 patients with IMN, 736 (70%) had normal thyroid function and 316 (30.0%) had abnormal thyroid function. The most common type of thyroid dysfunction was hypothyroidism (n = 226; 21.5%) including 212 cases of subclinical hypothyroidism (20.2%) and 14 cases of clinical hypothyroidism (1.3%). This was followed by NTIS (n = 79; 7.5%). In contrast, hyperthyroidism occurred less frequently (n = 11; 1.0%) (**Figure 1A**). When eGFR (unit: mL/min/1.73 m2) was >90, 60–90, and <60, the incidence of thyroid dysfunction was 26.1% (224/857), 45.9% (78/170), and 56.0% (14/25), respectively (**Figure 1B**).

Patients in the thyroid dysfunction group had a higher prevalence of nephrotic syndrome than those in the euthyroid group (68.4% versus 46.9%, *P* < 0.001). However, no significant variations in age, sex, course, or blood pressure were seen between the two groups (**Table 1**). Further subgroup analysis showed that, compared to the euthyroid group, the hypothyroid and NTIS groups had a higher incidence of nephrotic syndrome (**Table S1**).

**Table 1:** Clinicopathologic characteristics, treatment, and outcome of IMN patients without or with thyroid dysfunction before and after propensity score matching.

Characteristic	Unmatched data			Matched data		
	Euthyroidism (*n* = 736)	Thyroid dysfunction (n = 316)	*P* value	Euthyroidism (*n* = 282)	Thyroid dysfunction (*n* = 282)	*P* value
General Information						
Age (years)*	46.51 ± 12.63	46.43 ± 13.86	0.931	45.74 ± 12.62	46.66 ± 13.76	0.411
Male, *n* (%)	437 (59.4)	195 (61.7)	0.479	159 (56.4)	173 (61.3)	0.231
Course of disease (months)	1 (1, 4)	1 (1, 3)	0.317	1 (1, 4)	1 (1, 3)	0.303
Hypertension, *n* (%)	335 (45.5)	140 (44.3)	0.717	138 (48.9)	118 (41.8)	0.091
Systolic BP (mmHg)	133.96 ± 15.80	134.78 ± 17.59	0.456	134.74 ± 17.05	134.10 ± 17.32	0.658
Diastolic BP (mmHg)	85.60 ± 11.53	86.33 ± 11.57	0.348	86.40 ± 12.22	85.75 ± 11.33	0.513
Mean arterial pressure (mmHg)	101.72 ± 11.70	102.48 ± 12.21	0.341	102.51 ± 12.54	101.87 ± 11.88	0.530
Nephrotic syndrome, *n* (%)	345 (46.9)	216 (68.4)	< 0.001	184 (65.2)	184 (65.2)	1.000
Laboratory Result						
Blood urea nitrogen (mmol/L)	4.90 ± 1.64	5.23 ± 2.31	0.021	4.97 ± 1.89	5.12 ± 1.86	0.338
Serum creatinine (μmol/L)	67.70 ± 17.49	74.87 ± 25.13	< 0.001	70.32 ± 21.07	72.03 ± 18.85	0.311
Uric acid (μmol/L)	330.62 ± 88.85	320.81 ± 94.12	0.107	312.41 ± 79.67	324.86 ± 87.12	0.077
Albumin (g/L)	27.48 ± 6.35	23.36 ± 5.77	< 0.001	23.87 ± 5.16	24.07 ± 5.61	0.661
Total cholesterol (mmol/L)	6.79 (5.50, 8.25)	7.37 (6.09, 9.31)	< 0.001	7.38 (6.12, 9.31)	7.29 (6.05, 9.05)	0.465
Triglycerides (mmol/L)	1.99 (1.41, 2.99)	2.19 (1.52, 3.32)	0.017	2.02 (1.48, 3.12)	2.12 (1.43, 3.21)	0.613
eGFR (mL/min/1.73 m^2^)	104.13 ± 16.12	98.53 ± 20.24	< 0.001	101.93 ± 18.73	100.41 ± 18.18	0.328
White blood cell (×10^9^/L)	6.51 ± 1.88	6.65 ± 1.96	0.271	6.59 ± 1.96	6.61 ± 1.94	0.891
Hemoglobin (g/L)	134.71 ± 16.85	132.34 ± 18.05	0.041	131.47 ± 17.18	133.19 ± 17.63	0.241
Platelet (×10^9^/L)	239.03 ± 62.47	248.33 ± 75.70	0.055	239.07 ± 60.09	242.41 ± 67.56	0.535
Serum anti-PLA2R titer (RU/mL)	41.85 (10.95, 122.90)	86.25 (30.33, 215.05)	< 0.001	75.45 (21.75, 195.88)	79.05 (28.28, 203.23)	0.475
Serum anti-PLA2R titer > 50 RU/mL, *n* (%)	350 (47.6)	207 (65.5)	< 0.001	168 (59.6)	180 (63.8)	0.299
D-dimer (mg/L)	0.21 (0.11, 0.36)	0.29 (0.17, 0.58)	< 0.001	0.27 (0.16, 0.46)	0.26 (0.16, 0.51)	0.950
Proteinuria (g/d)	4.14 (2.25, 6.36)	5.56 (3.66, 8.25)	< 0.001	5.28 (3.17, 8.02)	5.25 (3.47, 7.89)	0.623
Renal Pathology						
Glomerulosclerosis, *n* (%)	440 (59.8)	197 (62.3)	0.436	178 (63.1)	175 (62.1)	0.794
Crescents, *n* (%)	47 (6.4)	26 (8.2)	0.281	23 (8.2)	22 (7.8)	0.877
Mesangial cell proliferation, *n* (%)	10 (1.4)	8 (2.5)	0.179	8 (2.8)	7 (2.5)	0.794
Tubular atrophy, *n* (%)	376 (51.1)	161 (50.9)	0.967	152 (53.9)	142 (50.4)	0.399
Interstitial fibrosis, *n* (%)	367 (49.9)	170 (53.8)	0.242	143 (50.7)	150 (53.2)	0.555
Inflammatory cell infiltration, *n* (%)	453 (61.5)	216 (68.4)	0.035	187 (66.3)	188 (66.7)	0.929
Arteriolar lesions, *n* (%)	488 (66.3)	222 (70.3)	0.210	175 (62.1)	196 (69.5)	0.062
Treatment						
RAASi, *n* (%)	488 (66.3)	186 (58.9)	0.021	168 (59.6)	167 (59.2)	0.932
Glucocorticoid or immunosuppressants alone, *n* (%)	341 (46.3)	133 (42.1)	0.205	122 (43.3)	120 (42.6)	0.865
Glucocorticoid and immunosuppressants, *n* (%)	259 (35.2)	148 (46.8)	< 0.001	134 (47.5)	131 (46.5)	0.800
Outcome						
Complete remission, n (%)	526 (71.5)	196 (62.0)	0.002	205 (72.7)	180 (63.8)	0.024
Relapse, n (%)	196 (28.2)	101 (34.6)	0.046	90 (33.8)	90 (34.5)	0.875
Composite endpoint, n (%)	55 (7.5)	35 (11.1)	0.055	19 (6.7)	33 (11.7)	0.042

Data were presented as means ± standard deviation, medians (interquartile range), or frequency (percentage). *The age range was 19-83 years old.

BP, blood pressure; eGFR, estimated glomerular filtration rate; PLA2R, phospholipase A2 receptor; RAASi, renin-angiotensin-aldosterone system inhibitor.

### Clinicopathological features and treatment before and after PSM

Patients in the thyroid dysfunction group exhibited higher levels of BUN, SCr, TG, TC, PLA2R antibody titer, D-dimer, and proteinuria and a greater percentage of PLA2R antibody titer > 50 RU/mL than those in the euthyroid group, but lower eGFR, albumin, and hemoglobin levels (all *P* < 0.05). Moreover, more patients in the thyroid dysfunction group had pathological changes in renal interstitial inflammatory cell infiltration (68.4% versus 61.5%, *P* = 0.035). Furthermore, the thyroid dysfunction group used a lower proportion of RAASi (58.9% versus 66.3%, *P* = 0.021) but a higher proportion of glucocorticoids combined with immunosuppressants (46.8% versus 35.2%, *P* < 0.001) (**Table 1**). Further subgroup analysis revealed that compared to the euthyroid group, the hypothyroid and NTIS groups had higher levels of SCr, TC, D-dimer, and PLA2R antibody titer, proteinuria, and a higher proportion of PLA2R antibody titer >50 RU/mL, but lower albumin and eGFR levels. In addition, the NTIS group had higher BUN and lower hemoglobin levels, while the hypothyroid group had higher levels of TG, platelets, and a higher proportion of glucocorticoids combined with immunosuppressants (**Tables S1**, **S2**).

After PSM, 282 pairs were successfully matched, including 282 cases in the euthyroid group and 282 cases in the thyroid dysfunction group. The baseline data of the two groups reached a balance (**Table 1**).

### Risk factors of thyroid dysfunction in IMN

The univariate logistic regression analysis showed that BUN, SCr, albumin, TC, TG, eGFR, hemoglobin, platelets, D-dimer, PLA2R antibody, proteinuria, and renal interstitial inflammatory cell infiltration were predictors of thyroid dysfunction in patients with IMN. Age, sex, and significant variables in the univariate analysis were included in the multivariate logistic regression. The results of the multivariate logistic regression revealed that female sex, lower albumin and eGFR levels, higher D-dimer level, and more proteinuria were independent risk factors for thyroid dysfunction in patients with IMN (**Table 2**).

**Table 2:** Risk factors of thyroid dysfunction in patients with IMN.

Characteristic	Univariate logistic		Multivariate logistic*	
	OR (95% CI)	*P* value	OR (95% CI)	*P* value
Sex (male vs female)	1.103 (0.841–1.455)	0.479	0.729 (0.540–0.985)	0.039
Age (years)	1.000 (0.989–1.010)	0.928		
Hypertension (yes vs no)	0.952 (0.730–1.241)	0.717		
Blood urea nitrogen (mmol/L)	1.095 (1.021–1.175)	0.011		
Serum creatinine (μmol/L)	1.017 (1.010–1.024)	0.000		
Uric acid (μmol/L)	0.999 (0.997–1.000)	0.108		
Albumin (g/L)	0.892 (0.870–0.914)	< 0.001	0.910 (0.885–0.935)	< 0.001
Total cholesterol (mmol/L)	1.148 (1.085–1.215)	< 0.001		
Triglycerides (mmol/L)	1.090 (1.013–1.173)	0.021		
eGFR (mL/min/1.73 m2)	0.982 (0.975–0.990)	< 0.001	0.990 (0.982–0.998)	0.017
White blood cell (×109/L)	1.039 (0.971–1.112)	0.271		
Hemoglobin (g/L)	0.992 (0.984–1.000)	0.041		
Platelet (×109/L)	1.002 (1.000–1.004)	0.039		
D-dimer (mg/L)	1.917 (1.474–2.493)	< 0.001	1.347 (1.044–1.738)	0.022
Serum anti-PLA2R titer (RU/mL)	1.001 (1.001–1.002)	< 0.001		
Proteinuria (g/d)	1.133 (1.092–1.176)	< 0.001	1.064 (1.021–1.108)	0.003
Glomerulosclerosis (yes vs no)	1.114 (0.849–1.460)	0.436		
Crescents (yes vs no)	1.314 (0.799–2.163)	0.282		
Mesangial cell proliferation (yes vs no)	1.886 (0.737–4.824)	0.186		
Tubular atrophy (yes vs no)	0.995 (0.764–1.295)	0.967		
Interstitial fibrosis (yes vs no)	1.171 (0.899–1.525)	0.242		
Inflammatory cell infiltration (yes vs no)	1.349 (1.020–1.785)	0.036		
Arteriolar lesions (yes vs no)	1.200 (0.902–1.597)	0.210		

*The values of multivariate logistic analysis were reported only for the statistically significant results.

OR, odd ratio; CI, confidence interval; eGFR, estimated glomerular filtration rate; PLA2R, phospholipase A2 receptor.

### Follow-up and prognosis analysis

**Figure 2:**
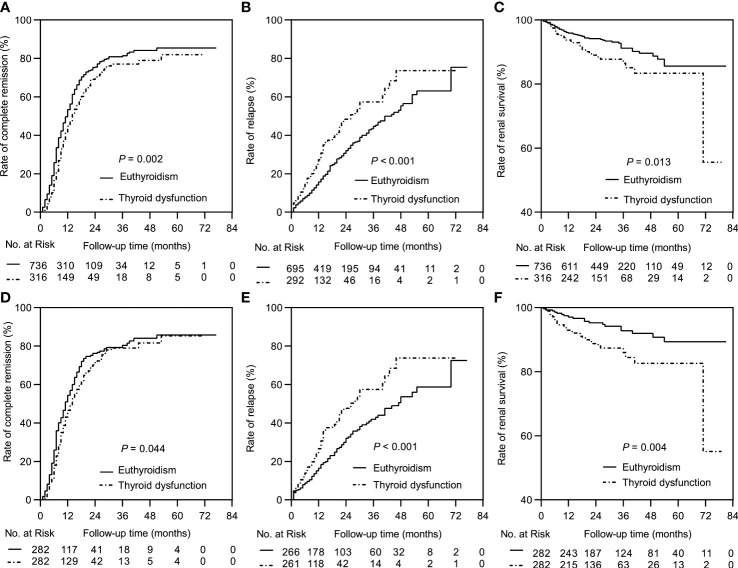
Kaplan-Meier curves between the euthyroid group and thyroid dysfunction group. In the complete dataset: **(A)** Complete remission rate; **(B)** Relapse rate; **(C)** Renal survival rate. In the propensity matched dataset: **(D)** Complete remission rate; **(E)** Relapse rate; **(F)** Renal survival rate.

**Figure 3:**
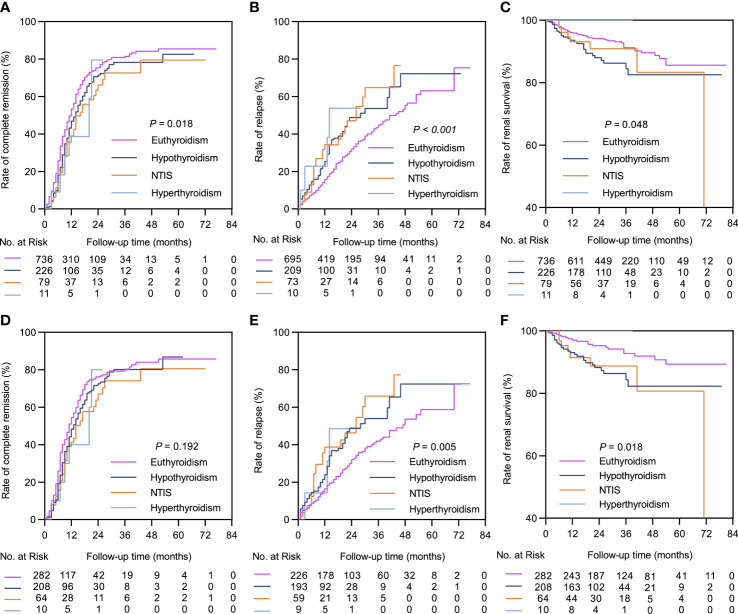
Kaplan-Meier curves between the euthyroid group and thyroid dysfunction subgroups. In the complete dataset: **(A)** Complete remission rate; **(B)** Relapse rate; **(C)** Renal survival rate. In the propensity matched dataset: **(D)** Complete remission rate; **(E)** Relapse rate; **(F)** Renal survival rate. NTIS, non-thyroid illness syndrome.

The median follow-up durations in the euthyroid and thyroid dysfunction groups were 28 (range =17-40) months and 24 (range = 14-34) months, respectively. The incidence rates of treatment response and composite events in the two groups are shown in **Table 1**. Results from the Kaplan-Meier curves revealed that the thyroid dysfunction group had a lower cumulative CR probability (*P* = 0.002, **Figure 2A**), higher cumulative relapse probability (*P* < 0.001, **Figure 2B**), and lower cumulative renal survival probability (*P* = 0.013, **Figure 2C**) than the euthyroid group. In the subgroup comparisons (**Figures 3**, **S1**), the thyroid dysfunction subgroups were separately compared with the euthyroid group. Kaplan-Meier curves identified a lower cumulative CR probability in the hypothyroid (*P* = 0.023) and NTIS groups (*P* = 0.017), but the differences were not statistically significant. Additionally, the cumulative relapse probabilities were higher in the hypothyroid (*P* < 0.001) and NTIS groups (*P* = 0.002), while the cumulative renal survival probability was lower in the hypothyroid group (*P* = 0.011). In the unadjusted model, Cox regression analysis revealed that thyroid dysfunction was a significant predictor of CR [hazard ratio (HR) = 0.781, *P* = 0.003], relapse (HR = 1.699, *P* = 0.001), and composite endpoint event (HR = 1.703, *P* = 0.014) in patients with IMN. After adjusting for age, sex, hypertension, proteinuria, albumin, eGFR, PLA2R antibody, and treatment, thyroid dysfunction remained an independent risk factor for IMN relapse (HR = 1.726, *P* < 0.001) and composite endpoint event (HR = 1.576, *P* = 0.043), but not for CR (HR = 0.941, *P* = 0.486) (**Table 3**).

**Table 3:** Cox regression analyses of complete remission, relapse, and composite endpoint event before propensity score matching.

	Unadjusted		Model 1^a^		Model 2^b^		Model 3^c^	
	HR (95% CI)	*P* value	HR (95% CI)	*P* value	HR (95% CI)	*P* value	HR (95% CI)	*P* value
Complete remission	0.781 (0.663–0.920)	0.003	0.777 (0.659–0.915)	0.003	0.934 (0.786–1.110)	0.439	0.941 (0.791–1.118)	0.486
Relapse	1.699 (1.259–2.292)	0.001	1.660 (1.231–2.240)	0.001	1.746 (1.291–2.360)	< 0.001	1.726 (1.277–2.333)	< 0.001
Composite endpoint event	1.703 (1.113–2.605)	0.014	1.716 (1.121–2.625)	0.013	1.507 (0.958–2.369)	0.076	1.576 (1.014–2.450)	0.043

a Model 1 was adjusted for age, sex and hypertension.

b Model 2 was adjusted for covariates in model 1 plus albumin, eGFR, proteinuria, and serum anti-PLA2R titer.

c Model 3 was adjusted for covariates in model 2 plus treatment strategies.

HR, hazard ratio; CI, confidence interval; eGFR, estimated glomerular filtration rate; PLA2R, phospholipase A2 receptor.

In the matched cohort, the median follow-up time in the euthyroid and thyroid dysfunction groups was 34 (20, 53) months and 24 (14, 34) months, respectively. The incidence rates of the different outcomes are also presented in **Table 1**. The thyroid dysfunction group had a lower cumulative CR probability (*P* = 0.044, **Figure 2D**), a higher cumulative relapse probability (*P* < 0.001, **Figure 2E**), and a poorer cumulative renal survival probability (*P* = 0.004, **Figure 2F**) than the euthyroid group. Subgroup analysis revealed that compared with the euthyroid group, the hypothyroid group (*P* = 0.003) and NTIS group (*P* = 0.004) had a higher cumulative relapse probability; the hypothyroid group (*P* = 0.006) had a lower cumulative renal survival probability, but differences in cumulative CR probability between the groups were not significant (**Figures 3**, **S2**). Results from the multivariate Cox regression analysis showed that after adjusting for the confounding factors, thyroid dysfunction remained an independent risk factor for CR (HR = 0.810, *P* = 0.045), relapse (HR = 1.721, *P* = 0.001), and composite endpoint event (HR = 2.113, *P* = 0.014) in patients with IMN (**Table 4**).

**Table 4:** Cox regression analyses of complete remission, relapse, and composite endpoint event after propensity score matching.

	Unadjusted		Model 1^a^		Model 2^b^		Model 3^c^	
	HR (95% CI)	*P* value	HR (95% CI)	*P* value	HR (95% CI)	*P* value	HR (95% CI)	*P* value
Complete remission	0.819 (0.671–1.001)	0.051	0.831 (0.679–1.016)	0.071	0.791 (0.644–0.970)	0.024	0.810 (0.659–0.996)	0.045
Relapse	1.699 (1.259–2.292)	0.001	1.657 (1.228–2.236)	0.001	1.726 (1.266–2.355)	0.001	1.721 (1.261–2.348)	0.001
Composite endpoint event	2.292 (1.292–4.066)	0.005	2.127 (1.194–3.787)	0.010	2.153 (1.192–3.888)	0.011	2.113 (1.165–3.831)	0.014

a Model 1 was adjusted for age, sex and hypertension.

b Model 2 was adjusted for covariates in model 1 plus albumin, eGFR, proteinuria, and serum anti-PLA2R titer.

c Model 3 was adjusted for covariates in model 2 plus treatment strategies.

HR, hazard ratio; CI, confidence interval; eGFR, estimated glomerular filtration rate; PLA2R, phospholipase A2 receptor.

## Discussion

In non-iodine deficient areas, the prevalence of hyperthyroidism, clinical hypothyroidism, and subclinical hypothyroidism is approximately 0.2%–1.3%, 1%–2%, and 4%–10%, respectively (18, 19). In our study (n= 1052), the prevalence of thyroid dysfunction was approximately 30%. Of this 30%, hypothyroidism accounted for 21.5% (subclinical hypothyroidism was about 20.2% and clinical hypothyroidism was about 1.3%), followed by NTIS (approximately 7.5%), while hyperthyroidism was low at about 1%. The prevalence of thyroid dysfunction was significantly higher in patients with IMN than in the general population, and the incidence increased with decreasing eGFR levels, as reported in the CKD cohorts (20). This may be related to several issues. First, massive proteinuria increases the excretion of carrier proteins, such as thyroid-binding globulin, transthyretin, and albumin. Second, the conversion of T4 to T3 is inhibited, and iodine clearance is also impaired. Finally, metabolic acidosis, inflammatory status, and diet are factors to consider (21, 22). The epidemiology of MN shows that it is more common in middle-aged and elderly people, with a 2:1 male predominance (3). This is reflected in our study sample of 1052 patients with IMN, in which the female-to-male ratio was 0.66:1.

This study also found that females, lower albumin and eGFR levels, higher D-dimer level, and severe proteinuria, were independent predictor variables for thyroid dysfunction in patients with IMN. Li et al. (10), in their cohort of 317 patients with nephrotic syndrome, found that SCr, TC, platelets, hemoglobin, albumin, and proteinuria were predictors of thyroid dysfunction. Li et al.’s findings are consistent with part of our results. The discrepancy may be related to different study samples and sizes between the two studies. Therefore, the thyroid functions of patients with IMN who have one or more of the risk factors should be monitored to avoid missed diagnoses and delayed treatment.

We found that patients in the thyroid dysfunction group had higher levels of SCr and proteinuria, and lower levels of albumin and eGFR. These factors have been identified as risk predictors of kidney disease progression, indicating that patients with IMN and thyroid dysfunction have more severe clinical manifestations (2). Thyroid hormones play an essential role in lipid metabolism, and thyroid dysfunction, particularly hypothyroidism, increases the likelihood of hyperlipidemia (23). Hyperlipidemia has been linked to an acceleration of renal function decline in patients with CKD (24). In this study, we discovered that patients with IMN and thyroid dysfunction tended to have higher levels of TC and TG, suggesting that thyroid dysfunction may induce more severe renal disorders by altering lipid levels. Research has also demonstrated that abnormal thyroid function increases the risk of anemia by affecting erythrocyte production and survival, together with iron metabolism and utilization (25). Moreover, anemia has been associated with poor prognosis in CKD (26). Furthermore, we observed that patients in the thyroid dysfunction group had a lower hemoglobin level, implying that thyroid dysfunction may negatively impact patients with IMN by reducing hemoglobin levels. Therefore, correcting the levels of blood lipids and hemoglobin in patients with IMN may be helpful.

PLA2R is the primary target antigen of IMN (70%–80%), and a PLA2R antibody titer greater than 50RU/mL is defined as a high risk for IMN according to the Kidney Disease Improving Global Outcomes (KDIGO) 2021 guideline. Additionally, a high level of PLA2R antibodies is an independent risk factor for persistent deterioration of renal function (2, 7, 27). Our study found that both the PLA2R antibody titer and the proportion of titers greater than 50 RU/mL were higher in the thyroid dysfunction group, inferring that thyroid dysfunction may have adverse effects on the development of IMN through an immune mechanism. Further investigations on this aspect are warranted.

Venous thromboembolism (VTE) is a potentially fatal complication of nephrotic syndrome, with the highest incidence in membranous nephropathy (7%–60%), and D-dimer is a vital biomarker for assessing VTE (28). Recent evidence suggests that thyroid hormones can affect the coagulation and fibrinolytic systems, increasing the risk of bleeding or thrombosis (29). Our study revealed that patients in the thyroid dysfunction group had a higher D-dimer level, implying that abnormal thyroid function may increase the risk of VTE in patients with IMN, which should be taken seriously in clinical practice. Regarding renal pathology, we found that patients in the thyroid dysfunction group demonstrated increased renal interstitial inflammatory cell infiltration relative to those in the euthyroid group, but there was no significant difference in glomerulopathy and arteriolar lesions. The investigation of thyroid dysfunction on pathological changes in patients with IMN is limited and requires further exploration.

Previous studies have reported that thyroid dysfunction increases the risk of cardiorenal injury and all-cause death in CKD (30–34). To date, there have been no large-scale clinical studies on thyroid dysfunction and the prognosis of IMN. In our large study cohort, patients with IMN combined with thyroid dysfunction demonstrated a poor prognosis, as well as severe clinical manifestations. Patients with thyroid dysfunction had a lower CR rate, a greater relapse rate, and a poorer kidney survival rate even after applying the PSM approach to minimize bias. The subgroup analysis further indicated that patients in the hypothyroid group had a higher relapse rate and a lower renal survival rate, whereas those in the NTIS group only had a higher relapse rate. There was no significant difference in the prognosis between the hyperthyroid and euthyroid groups, which may be due to the limited number of cases in our study. Notably, the multivariate Cox analysis confirmed that thyroid dysfunction was an independent risk factor for CR, relapse, and composite endpoint event in patients with IMN.

Thyroid dysfunction affects kidney function in several ways (21, 22). First, it can damage the renal structure, resulting in decreased kidney volume, thickened glomerular basement membrane, increased mesangial matrix, and capillary permeability. Second, thyroid hormone disorders alter eGFR through water-sodium metabolism, renal tubular ion transporters, and tubular-glomerular feedback. Thyroid dysfunction can also disrupt the autonomic regulation of renal blood perfusion *via* the renin-angiotensin-aldosterone system. Moreover, thyroid dysfunction influences cardiac output and blood volume by changing myocardial contractility, peripheral vascular resistance, and erythropoietin production, thereby altering renal blood flow. Consequently, patients with IMN and thyroid disorders should be regularly monitored. According to previous studies, thyroid hormone supplementation improves renal function in patients with thyroid deficiency, which may be related to thyroid hormone-enhanced circulating blood volume, renal blood flow, and endothelial function (35, 36). For patients with IMN and thyroid dysfunction, correcting abnormal thyroid function as soon as possible may be beneficial. Nevertheless, studies on the effectiveness and safety of thyroid hormone replacement therapy in patients with IMN are scarce. Hence, more basic research and multicenter cohort studies are required.

This study has some limitations. As a single-center retrospective study, the causal relationship between thyroid dysfunction and the prognosis of IMN could not be determined. The follow-up duration was also insufficient. Moreover, thyroid hormone levels were not dynamically observed during the follow-up period and patients with thyroid dysfunction were not treated or monitored regularly. We will continue to investigate this in future research.

In conclusion, using the PSM method in a large cohort of patients with IMN, this study found that patients with IMN and thyroid dysfunction have more severe clinical characteristics and worse prognoses, especially those with hypothyroidism. Moreover, thyroid dysfunction is an independent risk factor for poor prognosis in patients with IMN. Therefore, the thyroid function of patients with IMN should be monitored in clinical practice.

## Data availability statement

The original contributions presented in the study are included in the article/**Supplementary Material**. Further inquiries can be directed to the corresponding author.

## Ethics statement

The studies involving human participants were reviewed and approved by The Ethics Review Committee of the First Affiliated Hospital of Zhengzhou University (approval number: 2022-KY-1187-002). Written informed consent for participation was not required for this study in accordance with the national legislation and the institutional requirements.

## Author contributions

PW and JZ designed the study. YML, YCL, and HC collected the data. SW and BH analyzed the data. PW drafted the manuscript. All authors critically reviewed the article. All authors contributed to the article and approved the submitted version.
