# Supplementary material for: Clinicopathological features and prognosis of idiopathic membranous nephropathy with thyroid dysfunction
Source: Front Endocrinol (Lausanne). 2023 Mar 16;14:1133521. doi: 10.3389/fendo.2023.1133521 (PMC10060953; doi:10.3389/fendo.2023.1133521)
Supplement: Supplementary file 1 [file DataSheet_1.docx]

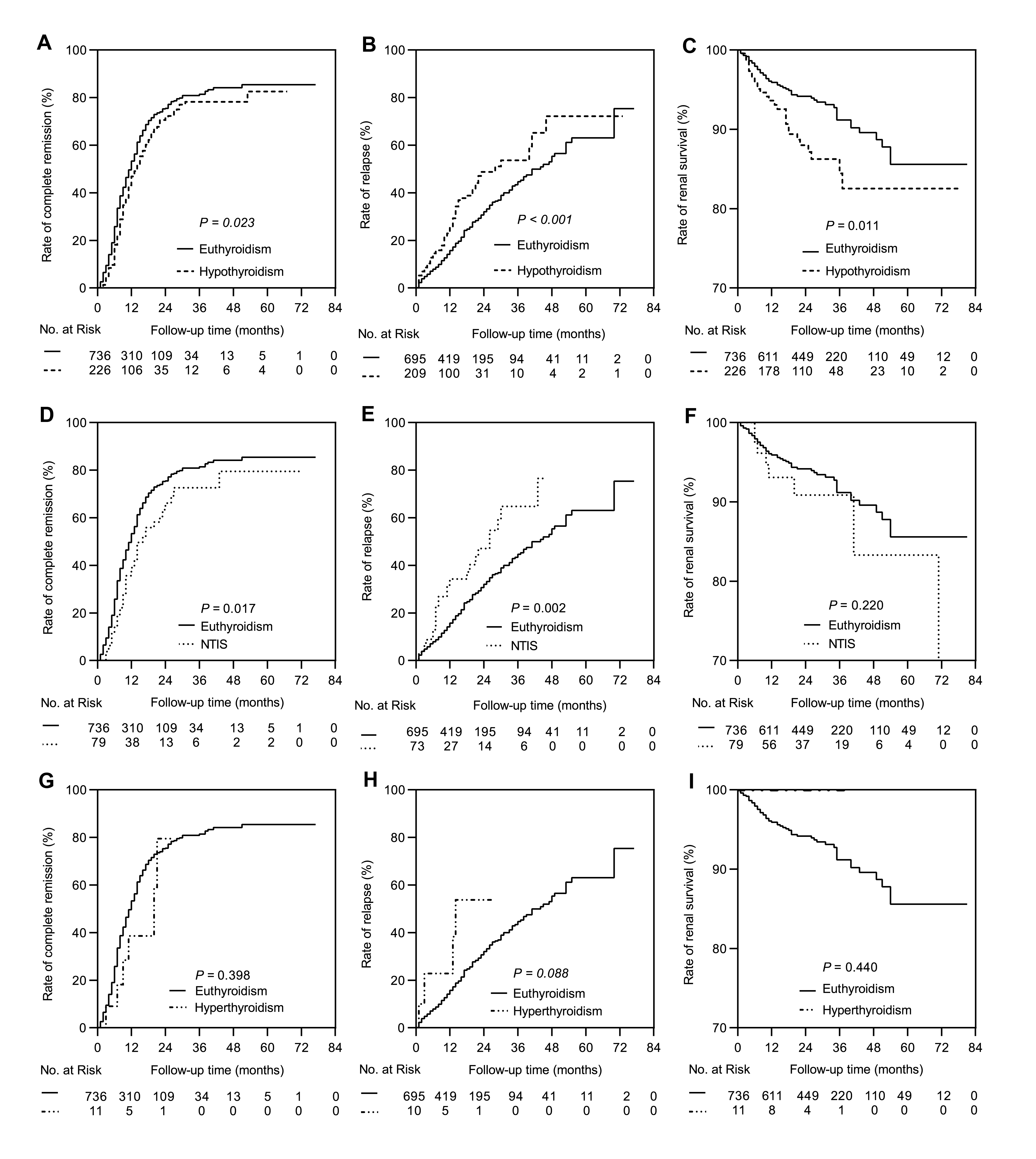


**Supplemental Figure 1** Kaplan-Meier curves before propensity matching. Comparison between hypothyroidism and euthyroidism groups: (A) Complete remission rate; (B) Relapse rate; (C) Renal survival rate. Comparison between NTIS and euthyroidism groups: (D) Complete remission rate; (E) Relapse rate; (F) Renal survival rate. Comparison between hyperthyroidism and euthyroidism groups: (G) Complete remission rate; (H) Relapse rate; (I) Renal survival rate. NTIS, non-thyroid disease syndrome.


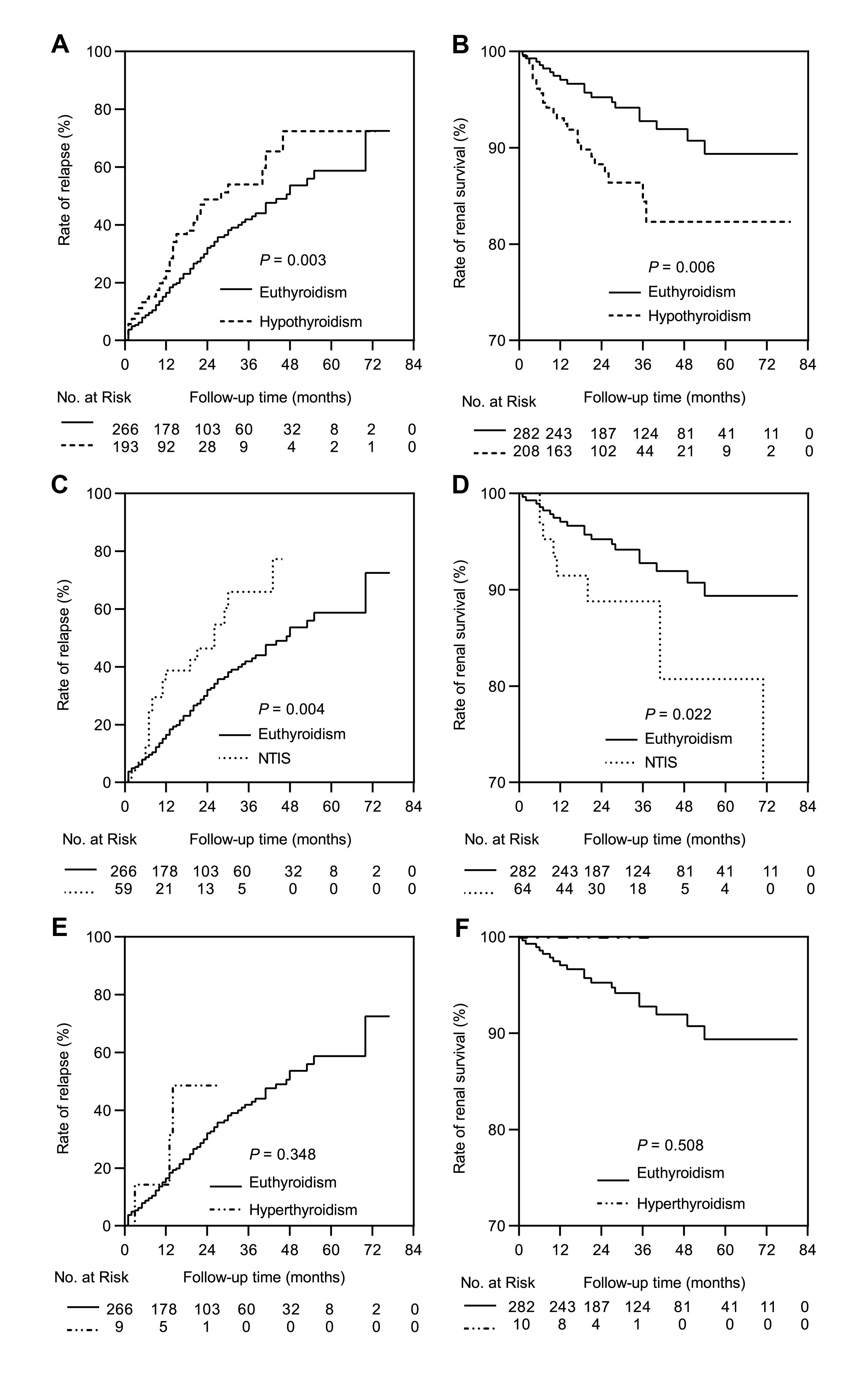


**Supplemental Figure 2** Kaplan-Meier curves after propensity matching. Comparison between hypothyroidism and euthyroidism groups: (A) Relapse rate; (B) Renal survival rate. Comparison between NTIS and euthyroidism groups: (C) Relapse rate; (D) Renal survival rate. Comparison between hyperthyroidism and euthyroidism groups: (E) Relapse rate; (F) Renal survival rate. NTIS, non-thyroid disease syndrome

**Supplemental Table 1** Clinicopathologic characteristics, treatment, and outcome of IMN patients with different thyroid function states before propensity matching

| Characteristic | Euthyroidism  (*n* = 736) | Hypothyroidism  (*n* = 226) | NTIS  (*n* = 79) | Hyperthyroidism  (*n* = 11) | *P* value |
| --- | --- | --- | --- | --- | --- |
| **General Information** |  |  |  |  |  |
| Age (years) | 46.51 ± 12.63 | 45.53 ± 13.16 | 49.65 ± 15.46 | 42.00 ± 13.05 | 0.065 |
| Male, *n* (%) | 437 (59.4) | 140 (61.9) | 46 (58.2) | 9 (81.8) | 0.430 |
| Course of disease (months) | 1 (1, 4) | 1 (1, 3) | 1 (1, 4) | 1 (1, 1) | 0.206 |
| Hypertension, *n* (%) | 335 (45.5) | 97 (42.9) | 39 (49.4) | 4 (36.4) | 0.705 |
| Systolic BP (mmHg) | 133.96 ± 15.80 | 134.70 ± 17.53 | 135.04 ± 18.16 | 134.64 ± 16.13 | 0.901 |
| Diastolic BP (mmHg) | 85.60 ± 11.53 | 86.28 ± 11.86 | 86.25 ± 10.73 | 88.00 ± 12.51 | 0.773 |
| Mean arterial pressure (mmHg) | 101.72 ± 11.70 | 102.42 ± 12.35 | 102.51 ± 11.84 | 103.55 ± 13.05 | 0.801 |
| Nephrotic syndrome, *n* (%) | 345 (46.9) | 153 (67.7) | 57 (72.2) | 6 (54.5) | < 0.001^ab^ |
| **Laboratory Result** |  |  |  |  |  |
| Blood urea nitrogen (mmol/L) | 4.90 ± 1.64 | 4.99 ± 1.66 | 5.88 ± 3.53 | 5.34 ± 2.08 | < 0.001^bd^ |
| Serum creatinine (μmol/L) | 67.70 ± 17.49 | 73.71 ± 22.22 | 77.84 ± 32.70 | 77.55 ± 18.40 | < 0.001^ab^ |
| Uric acid (μmol/L) | 330.62 ± 88.85 | 320.34 ± 84.89 | 319.27 ± 118.36 | 341.64 ± 84.45 | 0.361 |
| Albumin (g/L) | 27.48 ± 6.35 | 23.94 ± 5.82 | 21.46 ± 5.37 | 25.24 ± 4.77 | < 0.001^abd^ |
| Total cholesterol (mmol/L) | 6.79 (5.50, 8.25) | 7.34 (6.09, 9.15) | 7.70 (6.19, 9.55) | 7.29 (6.05, 9.29) | < 0.001^ab^ |
| Triglycerides (mmol/L) | 1.99 (1.41, 2.99) | 2.32 (1.57, 3.42) | 1.99 (1.27, 3.00) | 1.76 (1.29, 2.84) | 0.012^a^ |
| eGFR (mL/min/1.73 m^2^) | 104.13 ± 16.11 | 99.93 ± 19.92 | 94.18 ± 21.07 | 101.05 ± 17.54 | < 0.001^ab^ |
| White blood cell (×10^9^/L) | 6.51 ± 1.88 | 6.67 ± 1.76 | 6.58 ± 2.37 | 6.77 ± 2.78 | 0.708 |
| Hemoglobin (g/L) | 134.71 ± 16.85 | 134.24 ± 18.12 | 126.54 ± 17.43 | 135.00 ± 12.12 | 0.001^bd^ |
| Platelet (×10^9^/L) | 239.03 ± 62.47 | 257.12 ± 77.90 | 225.95 ± 67.28 | 228.36 ± 50.15 | < 0.001^ad^ |
| Serum anti-PLA2R titer (RU/mL) | 41.85 (10.95, 122.90) | 84.90 (30.15, 216.38) | 91.00 (30.40, 251.80) | 53.50 (20.60, 105.80) | < 0.001^ab^ |
| Serum anti-PLA2R titer > 50 RU/mL, *n* (%) | 350 (47.6) | 147 (65.0) | 54 (68.4) | 6 (54.5) | < 0.001^ab^ |
| D-dimer (mg/L) | 0.21 (0.11, 0.36) | 0.30 (0.16, 0.59) | 0.27 (0.17, 0.56) | 0.17 (0.09, 0.41) | < 0.001^ab^ |
| Proteinuria (g/d) | 4.14 (2.25, 6.36) | 5.60 (3.74, 7.96) | 6.09 (3.71, 9.00) | 4.49 (3.12, 8.00) | < 0.001^ab^ |
| **Renal pathology** |  |  |  |  |  |
| Glomerulosclerosis, *n* (%) | 440 (59.8) | 140 (61.9) | 51 (64.6) | 6 (54.5) | 0.786 |
| Crescents, *n* (%) | 47 (6.4) | 19 (8.4) | 6 (7.6) | 1 (9.1) | 0.745 |
| Mesangial cell proliferation, *n* (%) | 10 (1.4) | 7 (3.1) | 1 (1.3) | 0 (0) | 0.327 |
| Tubular atrophy, *n* (%) | 376 (51.1) | 117 (51.8) | 39 (49.4) | 5 (45.5) | 0.965 |
| Interstitial fibrosis, *n* (%) | 367 (49.9) | 125 (55.3) | 40 (50.6) | 5 (45.5) | 0.532 |
| Inflammatory cell infiltration, *n* (%) | 453 (61.5) | 158 (69.9) | 53 (67.1) | 5 (45.5) | 0.066 |
| Arteriolar lesions, *n* (%) | 488 (66.3) | 162 (71.7) | 52 (65.8) | 8 (72.7) | 0.472 |
| **Treatment** |  |  |  |  |  |
| RAASi, *n* (%) | 488 (66.3) | 135 (59.7) | 46 (58.2) | 5 (45.5) | 0.099 |
| Glucocorticoid or immunosuppressants alone, *n* (%) | 341 (46.3) | 93 (41.2) | 36 (45.6) | 4 (36.4) | 0.528 |
| Glucocorticoid and immunosuppressants, *n* (%) | 259 (35.2) | 109 (48.2) | 33 (41.8) | 6 (54.5) | 0.003^a^ |
| **Outcome** |  |  |  |  |  |
| Complete remission, *n* (%) | 526 (71.5) | 146 (64.6) | 44 (55.7) | 6 (54.5) | 0.009^b^ |
| Relapse, *n* (%) | 196 (28.2) | 70 (33.5) | 27 (37.0) | 4 (40.0) | 0.217 |
| Composite endpoint, *n* (%) | 55 (7.5) | 27 (11.9) | 8 (10.1) | 0 (0) | 0.127 |

Data were presented as means ± standard deviation, medians (interquartile range), or frequency (percentage). Abbreviation: BP, blood pressure; eGFR, estimated glomerular filtration rate; PLA2R, phospholipase A2 receptor; RAASi, renin-angiotensin-aldosterone system inhibitor; NTIS, non-thyroid disease syndrome.

^a^*P* < 0.05 between groups with hypothyroidism and euthyroidism.

^b^*P* < 0.05 between groups with NTIS and euthyroidism.

^c^*P* < 0.05 between groups with hyperthyroidism and euthyroidism.

^d^*P* < 0.05 between groups with hypothyroidism and NTIS.

**Supplemental Table 2** Clinicopathologic characteristics, treatment, and outcome of IMN patients with different thyroid function states after propensity matching

| Characteristic | Euthyroidism  (*n* = 282) | Hypothyroidism  (*n* = 208) | NTIS  (*n* = 64) | Hyperthyroidism  (*n* = 10) | *P* value |
| --- | --- | --- | --- | --- | --- |
| **General Information** |  |  |  |  |  |
| Age (years) | 45.74 ± 12.62 | 45.49 ± 13.06 | 51.13 ± 15.18 | 42.50 ± 13.64 | 0.014^bd^ |
| Male, *n* (%) | 159 (56.4) | 127 (61.1) | 38 (59.4) | 8 (80.0) | 0.394 |
| Course of disease (months) | 1 (1, 4) | 1 (1, 3) | 1 (1, 4) | 1 (1, 1.25) | 0.267 |
| Hypertension, *n* (%) | 138 (48.9) | 85 (40.9) | 30 (46.9) | 3 (30.0) | 0.245 |
| Systolic BP (mmHg) | 134.74 ± 17.05 | 134.09 ± 17.07 | 134.55 ± 18.81 | 131.6 ± 13.28 | 0.930 |
| Diastolic BP (mmHg) | 86.40 ± 12.22 | 85.79 ± 11.59 | 85.61 ± 10.73 | 85.70 ± 10.45 | 0.932 |
| Mean arterial pressure (mmHg) | 102.51 ± 12.54 | 101.89 ± 11.95 | 101.92 ± 11.99 | 101.00 ± 10.49 | 0.930 |
| Nephrotic syndrome, *n* (%) | 184 (65.2) | 136 (65.4) | 43 (67.2) | 5 (50.0) | 0.769 |
| **Laboratory Result** |  |  |  |  |  |
| Blood urea nitrogen (mmol/L) | 4.97 ± 1.89 | 4.93 ± 1.60 | 5.69 ± 2.44 | 5.44 ± 2.17 | 0.027^bd^ |
| Serum creatinine (μmol/L) | 70.32 ± 21.07 | 71.21 ± 18.87 | 73.67 ± 18.72 | 78.40 ± 19.16 | 0.421 |
| Uric acid (μmol/L) | 312.41 ± 79.67 | 324.91 ± 84.04 | 321.98 ± 97.25 | 342.20 ± 89.00 | 0.304 |
| Albumin (g/L) | 23.87 ± 5.16 | 24.42 ± 5.72 | 22.67 ± 5.18 | 25.80 ± 4.62 | 0.092 |
| Total cholesterol (mmol/L) | 7.38 (6.12, 9.31) | 7.26 (6.01, 8.96) | 7.35 (6.09, 9.43) | 7.26 (5.89, 8.36) | 0.848 |
| Triglycerides (mmol/L) | 2.02 (1.48, 3.12) | 2.24 (1.48, 3.37) | 1.95 (1.25, 2.68) | 1.69 (1.20, 2.36) | 0.069 |
| eGFR (mL/min/1.73 m^2^) | 101.93 ± 18.73 | 101.95 ± 18.03 | 95.52 ± 18.14 | 99.63 ± 17.80 | 0.074 |
| White blood cell (×10^9^/L) | 6.59 ± 1.96 | 6.65 ± 1.75 | 6.43 ± 2.38 | 7.06 ± 2.75 | 0.757 |
| Hemoglobin (g/L) | 131.47 ± 17.18 | 135.04 ± 17.75 | 126.84 ± 16.58 | 135.40 ± 12.69 | 0.006^d^ |
| Platelet (×10^9^/L) | 239.07 ± 60.09 | 250.65 ± 70.14 | 217.16 ± 54.22 | 232.80 ± 50.54 | 0.003^d^ |
| Serum anti-PLA2R titer (RU/mL) | 75.45 (21.75, 195.88) | 78.85 (28.63, 200.03) | 88.60 (25.83, 251.05) | 43.65 (19.15, 97.55) | 0.368 |
| Serum anti-PLA2R titer > 50 RU/mL, *n* (%) | 168 (59.6) | 131 (63.0) | 44 (68.8) | 5 (50.0) | 0.456 |
| D-dimer (mg/L) | 0.27 (0.16, 0.46) | 0.28 (0.16, 0.54) | 0.24 (0.16, 0.49) | 0.15 (0.09, 0.39) | 0.289 |
| Proteinuria (g/d) | 5.28 (3.17, 8.02) | 5.48 (3.58, 7.81) | 4.56 (3.13, 8.13) | 4.06 (3.10, 7.84) | 0.756 |
| **Renal pathology** |  |  |  |  |  |
| Glomerulosclerosis, *n* (%) | 178 (63.1) | 126 (60.6) | 43 (67.2) | 6 (60.0) | 0.801 |
| Crescents, *n* (%) | 23 (8.2) | 15 (7.2) | 6 (9.4) | 1 (10.0) | 0.939 |
| Mesangial cell proliferation, *n* (%) | 8 (2.8) | 6 (2.9) | 1 (1.6) | 0 (0) | 1.000 |
| Tubular atrophy, *n* (%) | 152 (53.9) | 105 (50.5) | 32 (50.0) | 5 (50.0) | 0.870 |
| Interstitial fibrosis, *n* (%) | 143 (50.7) | 111 (53.4) | 34 (53.1) | 5 (50.0) | 0.942 |
| Inflammatory cell infiltration, *n* (%) | 187 (66.3) | 140 (67.3) | 44 (68.8) | 4 (40.0) | 0.339 |
| Arteriolar lesions, *n* (%) | 175 (62.1) | 146 (70.2) | 42 (65.6) | 8 (80.0) | 0.218 |
| **Treatment** |  |  |  |  |  |
| RAASi, *n* (%) | 168 (59.6) | 123 (59.1) | 39 (60.9) | 5 (50.0) | 0.932 |
| Glucocorticoid or immunosuppressants alone, *n* (%) | 122 (43.3) | 88 (42.3) | 29 (45.3) | 3 (30.0) | 0.831 |
| Glucocorticoid and immunosuppressants, *n* (%) | 134 (47.5) | 98 (47.1) | 27 (42.2) | 6 (60.0) | 0.728 |
| **Outcome** |  |  |  |  |  |
| Complete remission, *n* (%) | 205 (72.7) | 137 (65.9) | 37 (57.8) | 6 (60.0) | 0.084 |
| Relapse, *n* (%) | 90 (33.8) | 64 (33.2) | 23 (39.0) | 3 (33.3) | 0.871 |
| Composite endpoint, *n* (%) | 19 (6.7) | 25 (12.0) | 8 (12.5) | 0 (0) | 0.127 |

Data were presented as means ± standard deviation, medians (interquartile range), or frequency (percentage). Abbreviation: BP, blood pressure; eGFR, estimated glomerular filtration rate; PLA2R, phospholipase A2 receptor; RAASi, renin-angiotensin-aldosterone system inhibitor; NTIS, non-thyroid disease syndrome.

^a^*P* < 0.05 between groups with hypothyroidism and euthyroidism.

^b^*P* < 0.05 between groups with NTIS and euthyroidism.

^c^*P* < 0.05 between groups with hyperthyroidism and euthyroidism.

^d^*P* < 0.05 between groups with hypothyroidism and NTIS.
